# Autophagy Promotes Porcine Parvovirus Replication and Induces Non-Apoptotic Cell Death in Porcine Placental Trophoblasts

**DOI:** 10.3390/v12010015

**Published:** 2019-12-20

**Authors:** Xiujuan Zhang, Yingli Xiong, Jie Zhang, Ting Shao, Songbiao Chen, Bichen Miao, Zhenyu Wang, Qian Du, Yong Huang, Dewen Tong

**Affiliations:** College of Veterinary Medicine, Northwest A&F University, Yangling 712100, China; cherlse2006@163.com (X.Z.); xylxiongyingli@126.com (Y.X.); jamiezhang07@126.com (J.Z.); shaotingsting@163.com (T.S.); chensongbiao2@126.com (S.C.); miaobichen@163.com (B.M.); zhenyuw2014nwsuaf@126.com (Z.W.); dewey3611@126.com (Q.D.)

**Keywords:** parvovirus, autophagy, replication, non-apoptotic cell death

## Abstract

Autophagy plays important roles in the infection and pathogenesis of many viruses, yet the regulatory roles of autophagy in the process of porcine parvovirus (PPV) infection remain unclear. Herein, we show that PPV infection induces autophagy in porcine placental trophoblasts (PTCs). Induction of autophagy by rapamycin (RAPA) inhibited the occurrence of apoptotic cell death, yet promoted viral replication in PPV-infected cells; inhibition of autophagy by 3-MA or ATG5 knockdown increased cellular apoptosis and reduced PPV replication. Interestingly, we found that in the presence of caspase-inhibitor zVAD-fmk, PPV induces non-apoptotic cell death that was characterized by lysosomal damage and associated with autophagy. Induction of complete autophagy flux by RAPA markedly promoted PPV replication compared with incomplete autophagy induced by RAPA plus bafilomycin (RAPA/BAF) in the early phase of PPV infection (24 h.p.i.). Meanwhile, induction of complete autophagy with RAPA increased lysosomal damage and non-apoptotic cell death in the later phase of PPV infection. Therefore, our data suggest that autophagy can enhance PPV replication and promote the occurrence of lysosomal-damage-associated non-apoptotic cell death in PPV-infected porcine placental trophoblasts.

## 1. Introduction

Porcine parvovirus (PPV), belongs to the genus Protoparvovirus of the family Parvoviridae, and is one of the major pathogens causing reproductive disorders in sow [1,2]. PPV is characterized as a small non-enveloped virus with a single-stranded DNA genome of about 5000 bases that contains the encoding genes of three major non-structural proteins (NS1, NS2, NS3), three structural proteins (VP1, VP2, VP3) and a late nonstructural protein (SAT) [3,4,5]. Like other autonomous parvoviruses, PPV structural proteins are responsible for the packaging of single-stranded viral progeny DNA to form infectious progeny virions [6,7,8], while non-structural proteins participate in the regulation of viral replication and its pathogenic process [9,10]. 

In recent years, numerous studies have demonstrated that autophagy plays a key role in the interactions of host cells with viruses [11,12]. Autophagy, as a dynamic, ubiquitous catabolic process to maintain cellular homeostasis and produce recycling energy, emerges as an important mechanism to regulate virus-host interaction when cells are faced with external stress stimuli [13,14]. However, depending on the intracellular environment, viruses and host cell types, the functional roles of autophagy in various viral infections differ [15]. Accumulating evidence suggests that autophagy works well beyond its original role as a turnover of cellular constituents. In response to various viral infections, autophagy modulates innate and adaptive immune responses via regulation of immune cell differentiation, survival, phagocytosis, antigen presentation, degranulation or cytokine production, which plays an important role in suppression or promotion of viral infection [16,17,18]. Notably, viruses have evolved various anti-elimination mechanisms through either employing the function of autophagy in different stages or by disturbing the antivirus function of autophagy. For example, classical swine fever virus (CSFV) uses its envelope protein E2 and nonstructural protein NS5A, which are co-localized with the membrane structure of autophagic vesicles to promote its replication [19], and hepatitis B virus (HBV) induces autophagy and consequently benefits for its replication [20]. Hence, autophagy can be seen a double-edged sword in the process of virus infection. Although the primary function of autophagy is to protect cells, sometimes it can contribute to cell death [21]. Persistent viral infection causes cellular homeostasis to lose control, resulting in the activation of the cell death pathway [22,23]. Recently, increasing lines of evidence have indicated that excessive autophagy is associated with cell death. When the stimulating factors inside or outside the cell persist, the level of autophagy rises in cells, which in turn aggravates cell damage and eventually causes cell death [24,25,26,27]. For example, cisatracurium induces excessive autophagy and significantly increases the cell death rate of mouse embryonic fibroblasts (MEFs) even in the presence of apoptosis inhibitor zVAD-fmk, but either 3-methyladenine (3-MA) treatment or ATG5 silence is able to reduce the cisatracurium-induced cell death [28]. Similarly, in parvoviruses, parvovirus H-1 (H-1PV) has been shown to kill human tumor cells in a non-apoptotic mechanism by inducing lysosomal permeabilization and promoting cathepsin release into cytosol [29,30]. However, the function and mechanism of autophagy in regulation of non-apoptotic cell death in the process of parvovirus infection remains undefined.

Despite autophagy having been implicated in the infection and pathogenic processes of different viruses including human parvovirus B19 [31], the roles of autophagy in porcine parvovirus (PPV) infection are poorly defined. In this study, we used PPV-infected placental trophoblast cells (PTCs) as a model to explore the roles of autophagy in the interaction of porcine parvoviruses with host cells. We found that PPV induces autophagy in PPV-infected cells to avoid apoptosis, which promotes the progeny virus replication in the early phase of infection, while in the later phase of infection PPV infection induces non-apoptotic cell death that is characterized by lysosomal damage and is associated with autophagy. 

## 2. Materials and Methods

### 2.1. Cell Culture and Virus Preparation

Placental trophoblast cells (PTCs) were isolated from healthy gilts and immortalized through reconstitution of telomerase in our previous study [32]. PTCs were cultured in Gibco Medium 199 (Cat No. 31100035, Gibco, USA) supplemented with 10% FBS (Cat No. 10099141, Gibco, USA), 100 U/mL of penicillin and 100 μg/mL of streptomycin (Cat No. P1400, Solarbio, China), at 37 °C in a 5% CO_2_ atmosphere incubator [32]. PPV China isolated strain (Genbank: MK993540) was isolated and stocked in our laboratory [33]. The PPV viruses were propagated in swine testis (ST) cells (ATCC, CRL-1746) or PK-15 (porcine kidney 15 cell line) that were donated from the Innovative team of animal pathogen surveillance and epidemiology in Harbin Veterinary Research Institute, CAAS. PPV was collected in the supernatant after cell lysis. The crude PPV preparation was purified using ultracentrifugation over sucrose cushions (2 mL of 50% sucrose plus 2 mL of 20% sucrose) by Optima XPN-100 Ultracentrifuge with a SW 41 Ti rotor, at 200,000× *g* for 2 h. Virus titers were determined by 50% tissue culture infective doses (TCID_50_) of assay in PTCs according to the Reed–Muench method, with the PPV China isolated strain at a titer of 10^6.32^ TCID_50_/0.1 mL.

### 2.2. Antibodies and Inhibitors

Antibodies against LC3 (Cat No. 12741), ATG5 (Cat No. 12994) and cathepsin D (Cat No. 2284) were purchased from Cell Signaling Technology (CST) (MA, USA). Anti-SQSTM1/p62 (ab101266) and anti-cathepsin L (ab103574) were purchased from Abcam PLC (Abcam, Cambridge, UK). Anti-β-actin (Cat No. A00702) was obtained from GenScript Biotech Corporation. Monoclonal anti-PPV capsid protein antibody was produced by 3C9 cell clones that were obtained from the American Type Culture Collection (Cat No. ATCC CRL-1745). Polyclonal anti-NS1 antibody was prepared by our laboratory, and was acquired from rabbits immunized with purified truncated NS1 protein expressed by pET32a vector in *Escherichia coli* (*E. coli*). Autophagy flux inhibitor Bafilomycin A1 (Cat No. 54645) was purchased from Cell Signaling Technology (CST) (MA, USA). Autophagy inhibitor 3MA (Cat No. 5142-23-4) was purchased from Sigma-Aldrich (USA).

### 2.3. Transmission Electron Microscopy

PTCs were mock infected or infected by PPV for 24 h at an multiplicity of infection (MOI) of 1, then cells were fixed with 2.5% glutaraldehyde and 4% paraformaldehyde in 0.1 M sodium phosphate buffer (pH 7.4) for 2 h at room temperature. The cells were harvested and fixed with 2.5% glutaraldehyde on ice for 2 h followed by post-fixation in 2% osmium tetroxide, then dehydrated with sequential washes in 50%, 70%, 90%, 95% and 100% ethanol. Areas containing cells were block mounted and thinly sliced. Sections were viewed under a transmission electron microscope (FEI Inc.). At least 15 cells were counted and the number of double-membrane vesicles was examined for each cell, as previously described [34]. Autophagosomes were defined as double-membrane vesicles measuring 0.2 to 1.0 μm.

### 2.4. Confocal Microscopy

For detection of autophagosomes, GFP-LC3 plasmid-transfected PTCs cells or RFP-GFP-LC3 adenovirus (both donated from Hai Zhang of Air Force Medical University)-infected PTCs cells were mock infected or infected with PPV for 24 h or treated with EBSS for 4 h. Cells were washed with PBS and fixed with ice-cold 4% (*wt*/*vol*) paraformaldehyde for 20 min at room temperature, then incubated with 0.1% Triton X-100 for 20 min, followed by indirect immunofluorescence detection using anti- PPV capsid antibodies (ATCC CRL-1745) and Alexa fluo647-conjugated anti-mouse IgG antibodies; nucleic acid was stained with 4, 6-diamidino-2-phenylindole (DAPI). Fluorescence was observed under a laser scanning confocal microscope (Leica, TCS SP8).

### 2.5. siRNA Transfection

ATG5-specific siRNA was synthesized from Sangon Biotech Co., Ltd. PTCs and seeded at a density of 2 × 10^5^ cells/well or 2 × 10^4^ cells/well in in 6- or 96-well plates, respectively. After adherent, cells were transfected with 50 nM ATG5 siRNA using Lipofectamine 2000 transfection reagent (Invitrogen, USA) according to the manufacturer’s instructions.

### 2.6. Quantitative PCR

The diluted plasmids containing PPV genomes were used as templates to draw the standard curve. Virus DNA was isolated from cells and cellular culture medium by proteinase K and SDS, and used for quantifying the copy numbers of the harvested viruses by real-time qPCR. The primers were: PPV-F: GGGGGAGGGCTTGGTTAGAATCAC; PPV-R: ACCACACTCCCCATGCGTTAGC.

### 2.7. Flow Cytometry Analysis

Cells were collected and stained with annexin V/PI or lysosomotropic dye LysoTracker Green DND26 (Invitrogen). The stained cells were analyzed by flow cytometry (BD Accuri C6), and the data were analyzed with flowJo V7.6 or flowJo V 10 software, as previously described [35].

### 2.8. Caspase Activity Assay

Caspase colorimetric assay kits were used to measure the activity of caspase-3 (KGA203, KeyGen Biotech, CHN) and caspase-9 (KGA403, KeyGen Biotech, CHN), respectively. According to the manufacturer’s recommendations, the collected cells were treated with lysis buffer, and protein concentration was measured using an Enhanced BCA Protein Assay Kit (P0010, Beyotime Institute of Biotechnology, CHN). Following this, 50-μL lysates (contain 200 μg protein) of each sample were loaded into microplates and incubated with each caspase substrate at 37 °C for 4 h, then the absorbance values of samples were measured at 405 nm in a microplate spectrophotometer (Infinite 200 PRO NanoQuant, Tecan, Switzerland).

### 2.9. Extraction of Cytosol

The release of cathepsin D and cathepsin L from lysosomes to the cytosol was detected by western blotting analysis of cytosol extracted using digitonin, as previously described [36]. Cells were incubated by rocking (100 rpm) on ice for 12 min in extraction buffer (250 mM sucrose, 20 mM HEPES, 10 mM KCl, 1.5 mM MgCl_2_, 1 mM Ethylene Diamine Tetraacetic Acid and 1 mM Ethylene Glycol Tetraacetic Acid) containing 25 mg/mL digitonin. The digitonin concentration and exposure time were optimized to result in the maximum release of cytosolic lactate dehydrogenase without disruption of lysosomes. The extraction buffer was withdrawn and proteins were precipitated by an addition of 5% trichloric acid. Proteins were pelleted and dissolved in lysis buffer (see western blotting protocol below) covering a 6-M urea, and the sample was neutralized by the addition of a 4-mL 1 M NaOH/100 mL sample.

### 2.10. Western Blotting

The cell pellets were lysed in RIPA with 1 mM Phenylmethanesulfonyl fluoride (PMSF) and protease inhibitors (Sigma) to collect cellular protein; culture supernatant was precipitated by 10% TCA/acetone to collect viral protein in culture supernatant. Similar amounts of protein from each extract were subjected to SDS-PAGE analysis and were transferred to polyvinyl difluoride (PVDF) membranes (Millipore). After blocking for 1 h with blocking buffer (5% nonfat milk and 0.1% Tween-20 in PBS), the membranes were incubated with the following primary antibodies at 4 °C overnight: anti-LC3, anti-PPV, anti-cathepsin D, anti-cathepsin L and anti-β-actin. Horseradish Peroxidase (HRP)-conjugated anti-mouse IgG or anti-rabbit IgG (Boster Biological Technology Co. Ltd) were used as secondary antibodies, and ECL (Bio-Rad) was used for chemiluminescent detection according to the manufacturer’s instructions. Image J was used to analyze and quantify the intensity of the protein band.

### 2.11. Statistical Analysis

All experiments were performed at least three times, and the results are representative of three or two of independent experiments. Data were presented as means ± SEM (SD). Multiple group data were analyzed by ANOVA and Bonferroni post-hoc tests, while comparisons between the two groups were performed by an unpaired Student’s *t* test. Statistical significance was defined as *p* < 0.05.

## 3. Results

### 3.1. PPV Infection Triggers the Accumulation of Autophagosomes

**Figure 1:**
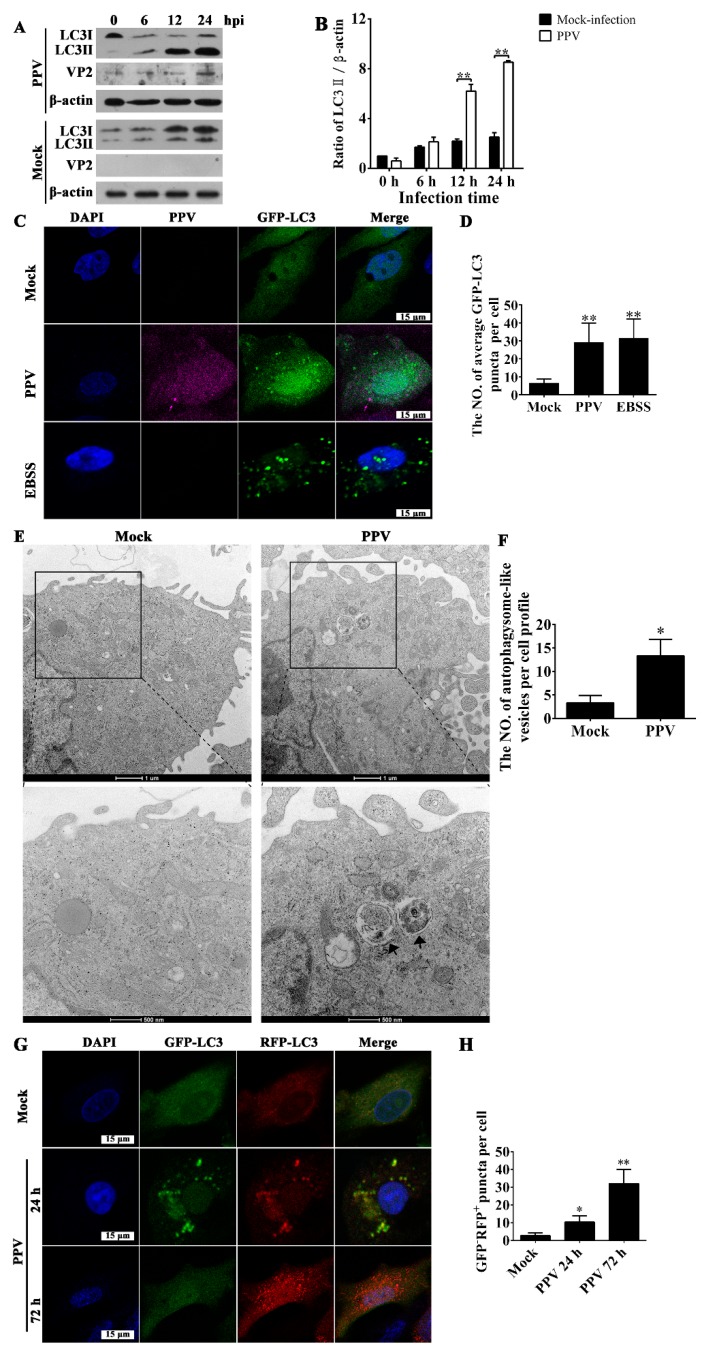
Porcine parvovirus (PPV) infection triggers the accumulation of autophagosomes. (**A**,**B**) Porcine placental trophoblasts (PTCs) were mock infected or infected with PPV. The cellular and viral proteins indicated were evaluated via western blotting (**A**), and the ratio of LC3-II/β-actin in PPV-infected cells was calculated and analyzed (**B**). (**C**,**D**) PTCs were transfected with GFP-LC3 vector and then mock infected or infected with PPV for 24 h, followed by indirect immunofluorescence detection using antibodies against PPV capsid (ATCC CRL-1745), and corresponding Alexa fluo647-conjugated secondary antibodies. GFP-LC3 puncta formation was then observed under laser scanning confocal microscopy (**C**). DAPI (blue) was used to stain nuclear DNA; scale bar: 15 μm. The number of GFP-LC3 puncta in each cell was counted, with at least 50 cells were counted for each group. Next, the average number of GFP-LC3 puncta per cell was calculated (**D**). (**E**,**F**) Mock-infected and PPV-infected PTCs were processed and analyzed for the accumulation of autophagosomes via transmission electron microscopy (**E**). Black arrows indicate autophagic vesicles; scale bar: 1 μm and 500 nm, respectively. The number of autophagosome-like vesicles per cell profile was counted, and at least 15 cells were included for each group (**F**). (**G**,**H**) PTCs were infected with adenovirus RFP-GFP-LC3 for 12 h, and then were infected with PPV. The formation of autophagosomes and autolysosomes were observed (**G**). The number of GFP^−^RFP^+^-LC3 puncta in each cell was counted for at least 50 cells for each group, and the average number of GFP^−^RFP^+^-LC3 puncta per cell was calculated (**H**). The results are mean ± SD of three experiments. * *p* < 0.05 versus the mock-infected cells; ** *p* < 0.01 versus the mock-infected cells.

To characterize the roles of autophagy in the PPV replication and interaction with host cells, we first sought to test whether PPV infection triggers the occurrence of autophagy. After PPV infection, VP2 protein maintained a relative lower level within 12 h post PPV infection, obviously increased at 24 h.p.i.; LC3-II levels markedly increased in porcine placenta trophoblast cells (PTCs) compared to mock-infected cells at 12 h and remained constant for 24 h, whereas the levels of LC3-I decreased with the increase of infection time (Figure 1A,B), suggesting that autophagosome formation cumulatively increases as PPV infection progresses. Puncta formation of GFP-LC3-labeled vesicles is regarded as another indicator of autophagosome formation. As shown in Figure 1C,D, PPV infection induced puncta formation of GFP-LC3-labeled vesicles in most PTCs transfected with GFP-LC3 plasmid, indicating that PPV infection indeed induces the accumulation of autophagosome. To confirm the existence of autophagosome in PPV-infected cells directly, we also performed ultrastructure analysis of cells using transmission electron microscopy (TEM). In mock-infected PTCs, autophagic vesicles were rarely observed (Figure 1E,F), whereas a large number of double-membraned autophagic vesicles containing wrapped cytoplasmic contents were accumulated in PPV-infected PTCs (Figure 1E,F). To further determine the characteristics of autophagy induced by PPV infection, we used a tandem reporter vector, RFP-GFP-LC3, in which GFP fluorescence is more sensitive to acidic pH and therefore will be attenuated in the acidic environment of lysosomes while RFP will not. In this case, the yellow fluorescent puncta will represent the formation of autophagosome, and the red fluorescent puncta represents autolysosome formation during autophagosome fusion with lysosome [34]. At 24 h.p.i., we observed a large amount of yellow fluorescent puncta in most of the PPV-infected cells, whereas large amounts of red fluorescent puncta were observed at 72 h.p.i. in most PPV-infected cells (Figure 1G,H), indicating that PPV mainly induces the formation of autophagosomes at the early phase of infection and further induces the formation of autophagic flux in the later phase of infection.

### 3.2. Autophagy Promotes PPV Replication

**Figure 2:**
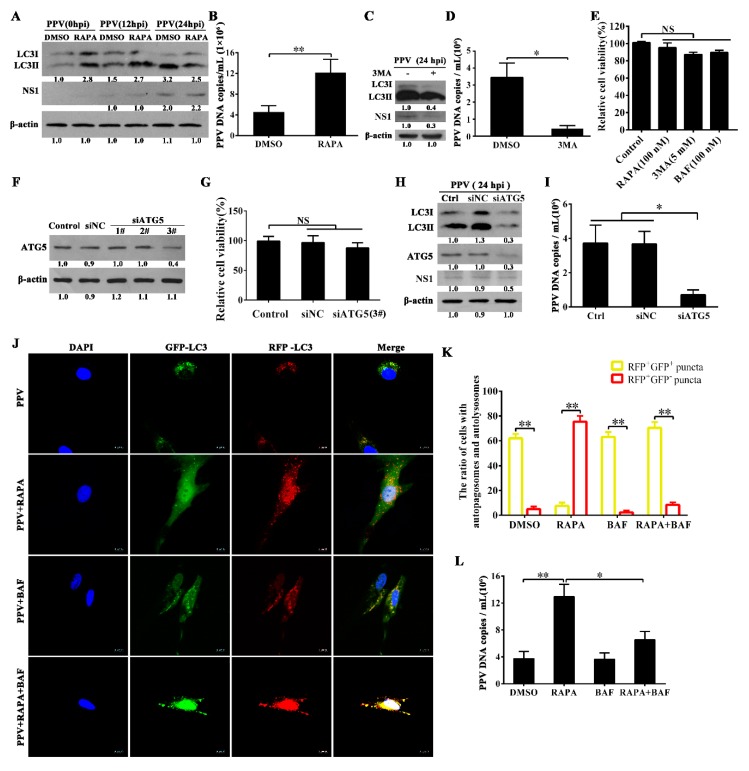
Autophagy promotes PPV replication. (**A**,**B**) PTCs were pretreated with solvent (DMSO) or rapamycin (RAPA, 100 nM) for 6 h, then infected with PPV (multiplicity of infection = 1). LC3 and PPV NS1 protein levels were evaluated by western blotting at 0, 12 and 24 h post-infection (**A**); PPV DNA copies were evaluated by Q-PCR at 24 h post-infection (**B**). (**C**,**D**) PTCs were pretreated with 3-MA (5 mM) for 4 h, then infected with PPV (MOI = 1) for 24 h, and LC3 and PPV NS1 were analyzed via western blotting (**C**). PPV DNA copies were measured by Q-PCR (**D**). (**E**) Cell viability was determined by MTT assay after treatments with the indicated concentration of rapamycin (RAPA), 3-methyladenine (3MA) or Bafilomycin A1 (BAF) for 24 h. (**F**,**G**) PTCs were transfected with siRNA duplexes against porcine ATG5 or nontargeting control siRNA for 48 h, cell lysates were used to evaluate ATG5 changes by western blotting (**F**). Cell viability was determined by MTT assay (**G**). (**H**,**I**) PTCs were transfected with ATG5 mRNA-specific siRNA (siATG5) or siRNA negative control (siNC) for 24 h, or untransfected (Ctrl), then infected with PPV for another 24 h. LC3, PPV NS1 and ATG5 were analyzed via western blotting (**H**), PPV DNA copies were measured by Q-PCR (**I**). (**J**,**K**) PTCs were infected with RFP-GFP-LC3 for 12 h then infected with PPV in the presence of RAPA (100 nM), BAF (100 nM) or RAPA (100 nM) plus BAF (100 nM). The formation of autophagosomes and autolysosomes was then observed (**J**), and the ratio of cells with autophagosomes (yellow puncta) or autolysosomes (red puncta) was counted and calculated in merge view (**K**); scale bar: 10 μm. The LSCM images show the quantification of autophagic vesicles by taking the average number of puncta in 50 cells. (**L**) PTCs were treated with solvent (DMSO), RAPA (100 nM), BAF (100 nM) or RAPA (100 nM) plus BAF (100 nM), then infected with PPV (MOI = 1) for 24 h, and PPV DNA copies were measured by Q-PCR. The results are mean ± SEM (SD) of three experiments. The data were analyzed by ANOVA followed by a Bonferroni’s post-hoc test or Student’s *t* test; * *p* < 0.05; ** *p* < 0.01.

We next wished to explore whether or not autophagy is involved in regulation of PPV replication. We first examined the effects of induction or inhibition of autophagy on the viral DNA copies. Rapamycin (RAPA), a well-known autophagy inducer [34], can significantly increase LC3-II levels in mock-infected PTCs, yet failed to markedly affect LC3-II levels in PPV-infected PTCs at 24 h.p.i. (Figure 2A). Intriguingly, RAPA pre-treatment significantly increased viral DNA copies in PPV-infected cells (Figure 2B). In contrast, 3-methyladenine (3-MA), a well-known autophagy inhibitor, markedly decreased LC3-II levels and viral DNA copies in PTCs infected with PPV (Figure 2C,D) without inducing toxic effects on cells at the indicated concentration (Figure 2E). Consistent with these observations, knocking down of the autophagy-related gene 5 (ATG5) by transfection of ATG5-specific siRNA (no significant effect on cell viability) inhibited the accumulation of LC3-II, resulting in reduced viral DNA copy numbers compared with control siRNA transfection in PTCs infected with PPV (MOI = 1) (Table 1, Figure 2F–I). These results suggest that induction of autophagy with RAPA promotes PPV replication, while inhibition of autophagy with 3-MA or ATG5 knockdown reduces the replication of PPV. 

To further identify the roles of accumulated autophagosomes in the replication of PPV, we determined the ratio of autophagic cells and the proportions of complete autophagic cells versus incomplete autophagic cells. In cells infected with PPV alone, yellow fluorescent LC3 puncta (autophagosome) appeared in about 60% cells, and nearly 5% cells showed red fluorescent LC3 puncta (autolysosome). By contrast, red fluorescent LC3 puncta appeared in about 76% of RAPA-pretreated cells infected with PPV, while nearly 6% cells showed yellow fluorescent LC3 puncta. In bafilomycin A1 (BAF)-pretreated cells infected with PPV, about 62% of cells showed yellow fluorescent LC3 puncta, while nearly 2% of cells showed red fluorescent LC3 puncta. In both RAPA- and BAF-pretreated cells infected with PPV, yellow fluorescent LC3 puncta appeared in about 72% of cells, while red fluorescent LC3 puncta appeared in 7% of cells (Figure 2J,K). RAPA pretreatment increased the incidence of autophagy over ~15%, while BAF treatment converted large numbers of cells from complete autophagy to incomplete autophagy in RAPA-pretreated cells infected with PPV. However, BAF did not appear to affect the proportion of complete autophagic cells and incomplete autophagic cells in cells infected with PPV (Figure 2J,K). In line with the difference of autophagy, RAPA pretreatment markedly increased viral DNA copies in PPV-infected cells regardless of the presence or absence of BAF, yet addition of BAF decreased the enhancement of viral DNA copies induced by RAPA in PPV-infected cells (Figure 2L), suggesting that induction of complete autophagy flux is more advantageous for PPV replication on the basis of autophagosome accumulation during PPV infection. 

### 3.3. Autophagy Blocks the Occurrence of Cell Apoptosis in PPV-Infected Cells

**Figure 3:**
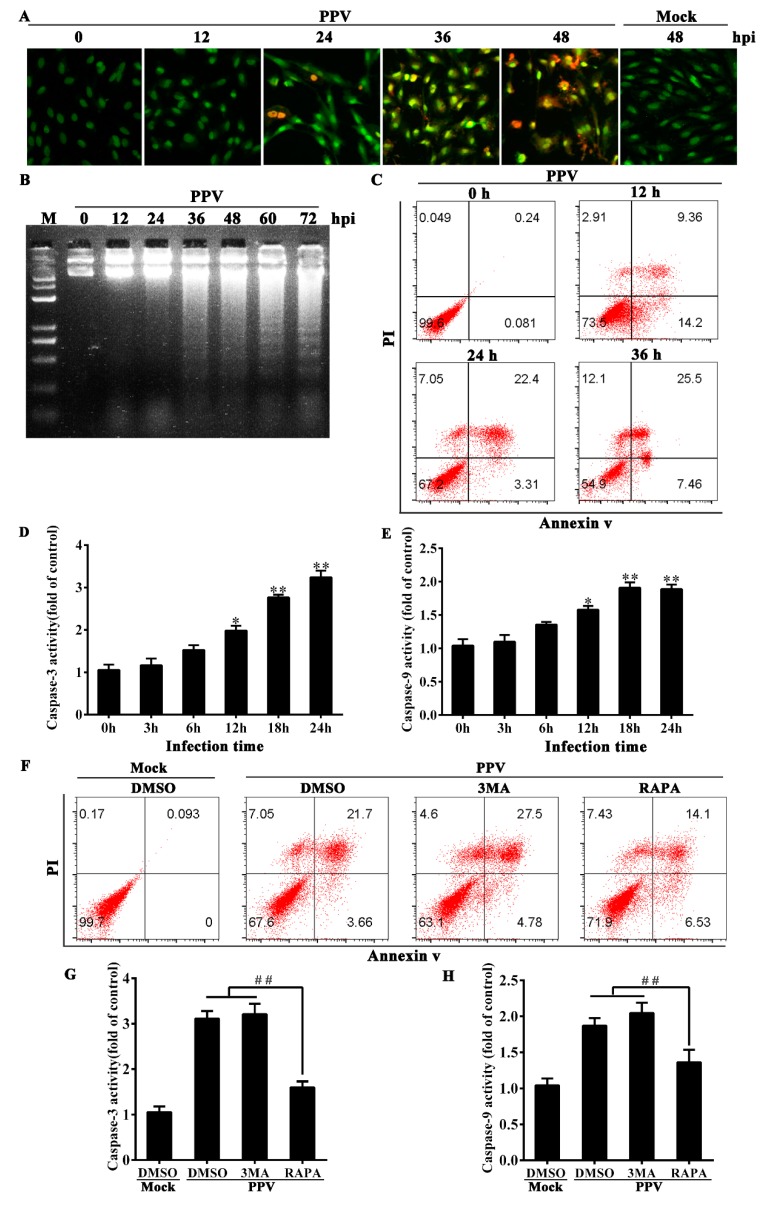
Induction of autophagy markedly decreases the occurrence of cell apoptosis in PPV-infected cells. (**A**) Morphological changes in PPV-infected cells. PTCs were infected with PPV at 1.0 MOI for the indicated times, with mock infection for 48 h serving as a control; samples were then stained with acridine orange/ethidium bromide (AO/EB, 100 μg/mL). (**B**) DNA fragmentation in PPV-infected cells. Cells were infected with PPV at 1.0 MOI for the indicated times. DNA was isolated and analyzed using agarose gel electrophoresis. Lane M: 100 bp DNA molecular weight marker. (**C**) Cell death analysis. PPV-infected cells were stained with annexin V and PI and analyzed by flow cytometry. (**D**,**E**) Measure of caspase-3 and caspase-9 activities at indicated times after PPV infection. (**F**–**H**) PTCs were treated with DMSO, 3MA or RAPA, then mock infected or infected with PPV for 24 h. (**F**) Flow cytometry analysis of annexin V/PI staining cells. (**G**,**H**) Measurement of caspase-3 and caspase-9 activities. The results are mean ± SEM of three experiments. ANOVA followed by Bonferroni’s post-hoc test; * *p* < 0.05; ** *p* < 0.01 versus the cells at 0 h. ^##^ *p* < 0.01.

It has been reported that PPV infection induces apoptosis in PK-15 cells [33]. In PTCs, we found that PPV infection also induced cell apoptosis as characterized by chromatin condensation (Figure 3A), chromosomal DNA fragmentation (Figure 3B), annexin V/PI-positive cells increase (Figure 3C), as well as caspase-9 and caspase-3 activation (Figure 3D,E). In order to clarify the relationship between autophagy and apoptosis in PPV-infected PTCs, we treated PPV-infected PTCs with an autophagy inducer or inhibitor to determine the changes of apoptosis rate and apoptosis-related proteins. The results of annexin V/PI staining showed that 1 MOI of PPV infection could induce apoptosis in ~25% of PTCs (annexin V-positive) at 24 h.p.i., while inhibition of autophagy with 3-MA modestly increased the percentage of annexin V-positive cells (32.01% ± 1.91%) in PPV-infected PTCs; however, RAPA-induced autophagy decreased annexin V-positive apoptotic cells by 5.12% ± 1.48 (Figure 3F). Consistent with these changes, addition of 3-MA modestly increased the levels of activated caspase-3 and caspase-9 in PPV-infected cells, whereas RAPA pretreatment markedly reduced the levels of activated caspase-3 and caspase-9 in PPV-infected cells (Figure 3G,H), suggesting that induction of autophagy can suppress the occurrence of apoptosis, while inhibition of autophagy promotes apoptosis. In addition, we observed 7.21% ± 1.15% of cells in PPV-infected PTCs to be annexin V-/PI+, and this portion of dead cells decreased in the presence of 3MA but increased in the presence of RAPA (Figure 3F), suggesting that PPV infection can induce other types of cell death associated with autophagy in addition to apoptosis. These results together indicate that PPV infection induces apoptotic cell death, autophagy and likely other types of non-apoptotic cell death, and that the induction of apoptosis is independent of autophagy yet, can be suppressed by autophagy.

### 3.4. PPV Can Induce Non-Apoptotic Death in PTCs Characteristic of Lysosome Damage

**Figure 4:**
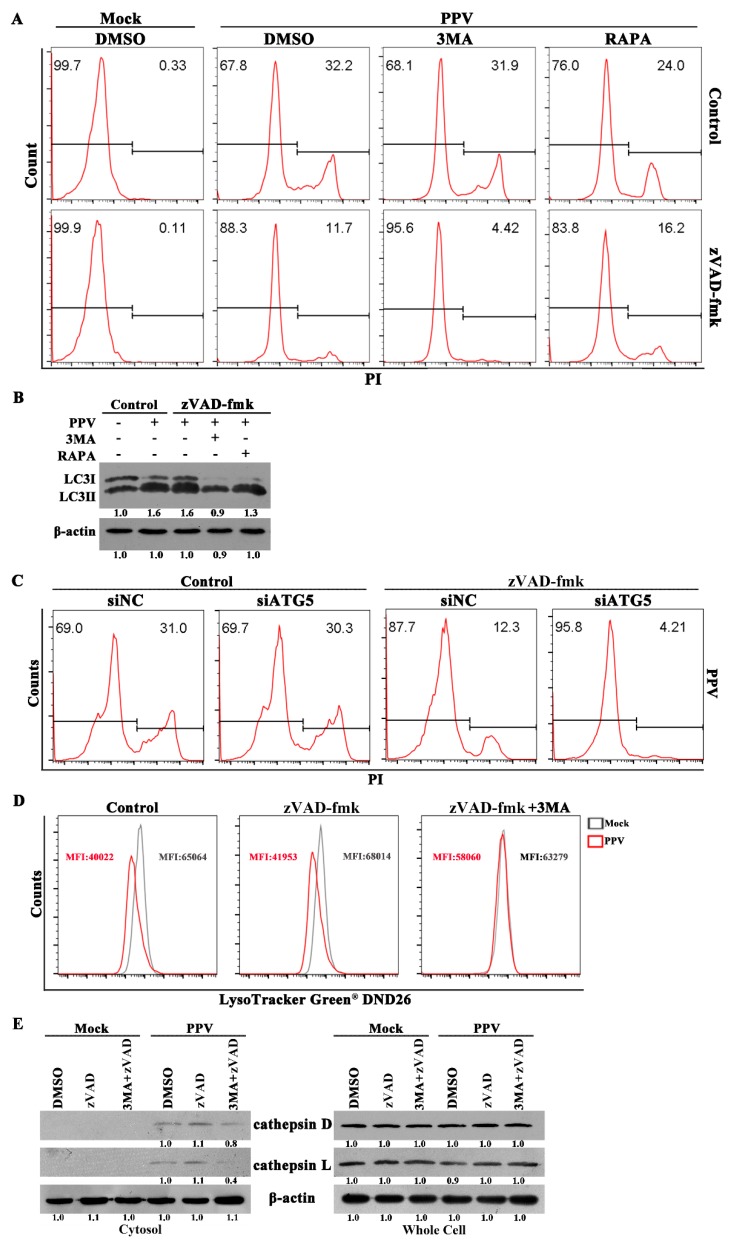
PPV infection can induce non-apoptotic cell death in PTCs. (**A**) PTCs were treated with DMSO, 3MA or RAPA, then mock infected or infected with PPV for 24 h in the presence or absence of zVAD-fmk. Flow cytometry analysis of death cells (PI staining cells). (**B**) Analysis of LC3 change in the presence of zVAD-fmk. (**C**) Knockdown of ATG5 decreases cell death in the presence of zVAD-fmk. PTCs were transfected with ATG5-specific siRNA (siATG5) or siRNA negative control (siNC) for 24 h, and then infected with PPV for another 24 h in the presence of or absence of zVAD-fmk. Cells were stained with PI and analyzed by flow cytometry. (**D**,**E**) PPV infection causes the decline of lysosome tracker DND26 and the release of lysosomal proteases (cathepsin D and L) from lysosome, which is blocked by autophagy inhibitors. PTCs were treated with DMSO, zVAD-fmk or zVAD-fmk plus 3MA, then mock infected or infected with PPV. DND26 was measured (24 h.p.i.) by flow cytometry (**D**), and cytosol cathepsin D and L were detected (72 h.p.i.) by western blotting (**E**). Results are representative of three independent experiments.

To further determine whether non-apoptotic death was present in PPV-infected cells, we treated PTCs with specific caspase inhibitors to block the occurrence of apoptosis, then measured the cell death of PPV-infected cells in the presence of autophagy inducer (RAPA) or inhibitor (3-MA). Caspase-inhibitor zVAD-fmk decreased PI-positive cells by ~20%, but PI-positive cells still remained at about 10% in the presence of zVAD-fmk (Figure 4A). Addition of 3-MA to inhibit PPV-induced autophagy did not alter the rate of PI-positive cells in the absence of zVAD-fmk, but further decreased PI-positive cells by ~6%, leaving the remaining 5% cells PI-positive in the presence of zVAD-fmk (Figure 4A,B). Conversely, although the addition of RAPA to promote PPV-induced autophagy decreased PI-positive cells by ~8% in the absence of zVAD-fmk, RAPA treatment increased PI-positive cells by ~5% in the presence of zVAD-fmk (Figure 4A,B). Consistent with these results, knockdown of autophagy gene ATG5 also apparently reduced PI-positive cells by ~8% in zVAD-fmk-treated cells 24 h after PPV infection (Figure 4C). These results indicate that additional forms of cell death exist in PPV-infected cells that are independent of apoptosis, yet upregulated by autophagy.

To further determine the biochemical features of this type of cell death in PPV-infected cells, we used lysosome tracker DND-26 and lysosomal proteases (cathepsin D and L) to monitor lysosomal membrane permeabilization of PPV-infected cells. Lysosome tracker DND-26 is accumulated in acidic organelles in live cells and released from lysosomes when lysosomal membrane permeabilization increases or lysosomes are damaged [37]. Compared with mock infection, PPV infection markedly decreased the mean fluorescence intensity (MFI) of DND-26 (Figure 4D). Presence of zVAD-fmk did not alter the decrease of DND-26 fluorescence induced by PPV infection, but addition of autophagy inhibitor (3-MA) efficiently blocked the decline of DND-26 fluorescence (Figure 4D). Similar to DND-26 fluorescence changes, PPV infection promoted the release of cathepsin D and cathepsin L from lysosome to cytosol, which were blocked by 3-MA but not by zVAD-fmk (Figure 4E). Altogether, these data demonstrate that PPV infection can induce non-apoptotic death characterized by lysosomal damage in PTCs and associated with autophagy.

### 3.5. Autophagy Flux Increases the Ratio of Non-Apoptotic Cell Death in the Later Phase of PPV Infection

**Figure 5:**
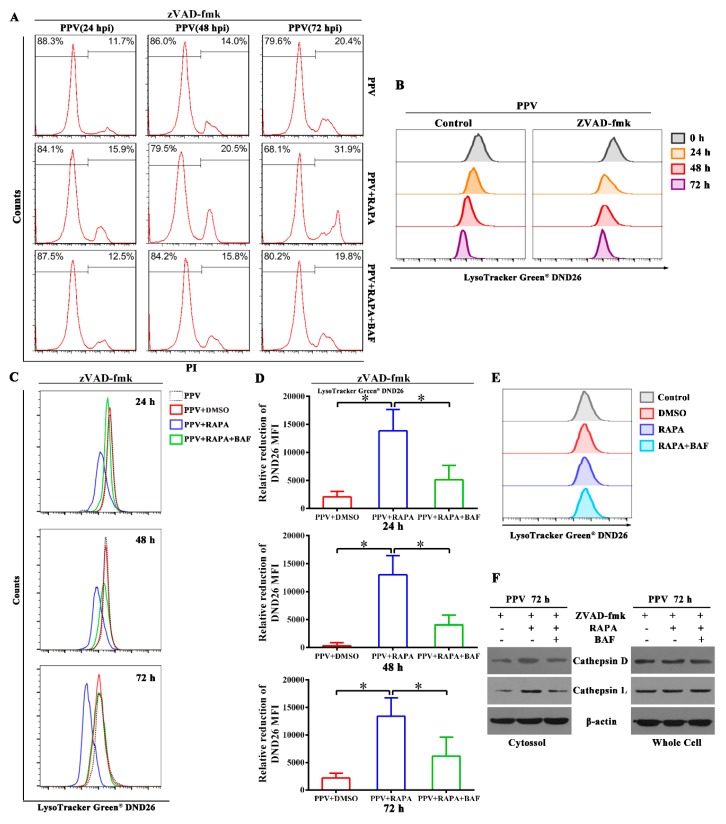
Autophagy flux increases the ratio of non-apoptotic cell death in the later phase of PPV infection. (**A**) PTCs were treated with RAPA (100 nM) alone or RAPA (100 nM) plus BAF (100 nM), then infected with PPV in the presence of zVAD-fmk. Cells were stained with PI and analyzed by flow cytometry at the indicated times after PPV infection. (**B**) PTCs were infected with PPV in the presence or absence of zVAD-fmk, then stained by LysoTracker Green DND26 to evaluate lysosomal membrane permeabilization at the indicated times post-PPV infection. (**C**,**D**) Incomplete autophagy causes less lysosome damage than complete autophagy in PPV-infected cells. PTCs were treated as in (**A**) and stained by LysoTracker Green DND26 to evaluate lysosomal membrane permeabilization (**C**). The relative reduction value of mean fluorescence intensity (MFI) of DND26 in the indicated cells (DND26 MFI of PPV-infection cells –DND26 MFI of indicated cells) was calculated and analyzed statistically (**D**). (**E**) PTCs were treated with RAPA (100 nM) alone or both RAPA (100 nM) and BAF (100 nM), then stained by LysoTracker Green DND26 to evaluate lysosomal membrane permeabilization. (**F**) PTCs were treated as in (**A**) and cytosol cathepsins D and L were detected (72 h.p.i.) by western blotting. The results are mean ± SEM (SD) of three experiments. ANOVA followed by Bonferroni’s post-hoc test, or Student’s *t* test, * *p* < 0.05; ** *p* < 0.01.

As presented earlier, PPV infection can induce autophagy in host cells. To gain insight into the roles of autophagy in the infection process of PPV, we interrogated the functions of autophagy in the survival of host cells. We used RAPA to induce complete autophagy and used RAPA plus BAF to achieve incomplete autophagy in PPV-infected cells. In the presence of apoptosis inhibitor zVAD-fmk, the percentage of cell death (PI-positive) continued to increase with time up to 72 h (the last time-point tested) (Figure 5A). Addition of RAPA increased the rate of PI-positive cells at 24, 48 and 72 h.p.i., particularly at later time points, but addition of RAPA together with BAF failed to increase the percentage of dead cells relative to PPV infection alone (Figure 5A). Further analysis of lysosomal membrane permeabilization by lysosome tracker DND-26 showed that DND-26 fluorescence continued to decrease with PPV infection time, suggesting that lysosomal membrane permeabilization increased with infection time (Figure 5B). Addition of RAPA apparently decreased the intensity of DND-26 fluorescence at 24, 48 and 72 h.p.i., whereas addition of RAPA/BAF restored the intensity of DND-26 fluorescence relative to RAPA treatment in the presence of apoptosis inhibitor zVAD-fmk (Figure 5C,D). However, addition of RAPA alone or RAPA/BAF did not affect the intensity of DND-26 fluorescence in the cells without PPV infection (Figure 5E). These results suggest that addition of RAPA-induced complete autophagy contributes to lysosome damage compared with RAPA/BAF-induced incomplete autophagy in PPV-infected cells. In agreement with the differences in cell survival, the release of cathepsin D and cathepsin L from lysosome to cytosol at 72 h.p.i. was markedly decreased in the PPV-infected cells with or without RAPA/BAF than in PPV-infected cells with RAPA alone (Figure 5F). Together, these results demonstrate that autophagy flux increases the ratio of non-apoptotic cell death in the later phase of PPV infection.

## 4. Discussion

Autophagy is a cellular catabolic process conserved in all eukaryotes that plays important roles in the interaction of host cells with viruses [11,12]. In this study, we demonstrated that PPV infection induces cell autophagy, apoptosis and non-apoptotic cell death. In PPV-infected cells, autophagy flux induced by RAPA could suppress the occurrence of cell apoptosis and promote virus replication, while inhibition of autophagy with 3-MA or ATG5 knockdown increased cell apoptosis and reduced PPV replication. In the presence of caspase-inhibitor zVAD-fmk, PPV could induce non-apoptotic cell death characterized by lysosomal damage in PTCs. Interestingly, although induction of autophagy (complete/incomplete) is beneficial for PPV replication in the early phase of PPV infection, only induction of complete autophagy flux increases the ratio of non-apoptotic cell death in the later phase of PPV infection.

In recent years, autophagy has been reported to be involved in the regulation of viral infections. For example, the autophagic process is required for balancing HCV-host cell interactions and is involved in the pathogenesis of HCV-related chronic liver diseases; furthermore, HCV is able to induce an autophagic response and inhibit the fusion of autophagosomes with lysosomes [38,39]. Similarly, suppression of pancreatic acinar cell-specific autophagy induced by CVB3 reduces viral replication and pathogenesis in vivo [40]. Similar to HCV and CVB3, PPV infection induces a large number of cells to form autophagy in the early phase of PPV infection, and induction of autophagy by RAPA promotes viral replication, while inhibition of autophagy by 3-MA or ATG5 knockdown significantly reduces viral replication. 

Cell death, as a direct consequence of viral infection, contains three major morphological types: apoptosis (type I cell death), autophagic cell death (type II) and necrosis (type III). Autophagy is intimately associated with eukaryotic cell death. In some cases, the same proteins can regulate both autophagy and apoptosis. Apoptotic signals can regulate autophagy, and autophagic signals are also involved in the regulation of apoptosis (and most likely other cell death mechanisms) [41,42,43]. For instance, the autophagy triggered by the virus may protect chronic HCV-infected cells from stress-induced apoptosis, which promotes cell survival [39]. For parvovirus, Di Piazza et al. reported that H-1PV is able to kill cells through a non-apoptotic lysosomal mechanism in addition to the apoptotic mechanism [29]. Recently, their further study showed that H-1PV kills human tumor cells by inducing lysosomal permeabilization and cathepsin release into the cytosol [30]. Similarly, PPV infection also appears to trigger non-apoptotic death characterized by lysosomal permeabilization and cathepsin release into the cytosol, which is associated with autophagic flux in PPV-infected PTCs. Our experiments also showed that PPV infection induces cell autophagy, apoptosis and non-apoptotic cell death simultaneously. In PPV-infected cells, induction of autophagy with RAPA could inhibit the occurrence of cell apoptosis, while inhibition of autophagy with 3-MA or ATG5 knockdown increased cell apoptosis. These results indicate that PPV induction of apoptosis is independent from autophagy, yet inhibited by autophagy in PPV-infected cells. 

**Figure 6:**
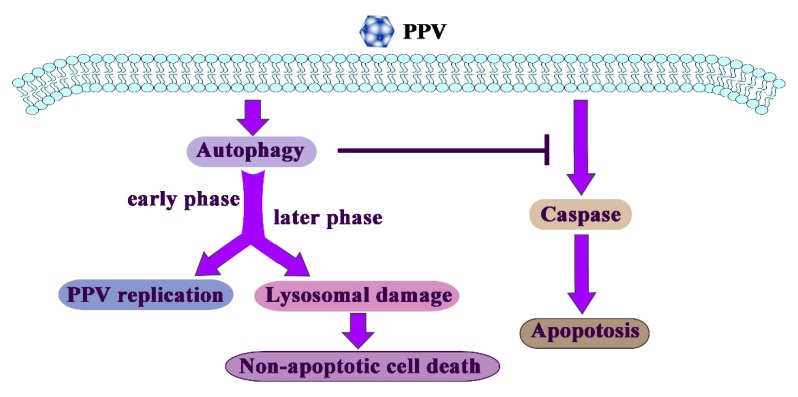
Model of autophagy action during PPV infection. PPV infection induces cell autophagy, apoptosis and non-apoptotic cell death. Induction of apoptosis is independent from autophagy, yet suppressed by autophagy. Autophagy is rapidly elevated and acts primarily as a survival mechanism, but further forming autophagy flux may also lead to non-apoptotic cell death in the later phase of infection, which is characterized by lysosomal damage in PPV-infected cells.

In response to pathogen infection, autophagy is rapidly elevated and acts primarily as a survival mechanism, but this may also lead to demise of the cells in the long-term. PPV infection can increase non-apoptotic cell death in the later phase of PPV infection, which is independent of apoptosis yet inhibited by autophagic inhibitors or induced by autophagic inducers, and is characterized by lysosomal damage in PTCs (Figure 6). Overall, the results presented here provide strong evidence that non-apoptotic cell death occurs during PPV infection, and reveal a critical role of autophagy in the PPV infection induction of non-apoptotic PTCs death.

## Figures and Tables

**Table 1:** Sequences of double-stranded RNA used to specifically ablate ATG5 expression in PTCs.

siRNA	Sense (5′-3′)	Antisense (5′-3′)
ATG5-1#	CCCUCUAUCAGGAUGAGAUTT	AUCUCAUCCUGAUAGAGGGTT
ATG5-2#	GGAUGUAAUUGAAGCUCAUTT	AUGAGCUUCAAUUACAUCCTT
ATG5-3#	CCAUCAACCGGAAACUCAUTT	AUGAGUUUCCGGUUGAUGGTT
Non-targeting siNC	UUCUCCGAACGUGUCACGUTT	ACGUGACACGUUCGGAGAATT
